# The European Society of Pediatric Nephrology Board Examination—a certified benchmark of knowledge and clinical practice with global recognition

**DOI:** 10.1007/s00467-026-07436-x

**Published:** 2026-06-30

**Authors:** Ana Teixeira, Stella Stabouli, Rezan Topaloglu, Aysun Karabay Bayazit, Olivia Boyer, Dorota Drozdz, Rukshana Shroff, Martin Christian, Zainab Arslan, Justine Bacchetta, Jerome Harambat, Nikoleta Printza, Louise Oni, Daniella Levy Erez, Caroline Platt, Dieter Haffner, Jun Oh, Elena Levtchenko

**Affiliations:** 1 Pediatric Nephrology Department, Centro Materno-Infantil Do Norte, Unidade Local de Saúde de Santo António, Largo da Maternidade de Júlio Dinis, 4050-651 Porto, Portugal; 2 Department of Paediatrics, Aristotle University Thessaloniki, Hippokratio Hospital, Thessaloniki, Greece; 3 Department of Pediatrics, Hacettepe University School of Medicine, Ankara, Turkey; 4 Acibadem Healthcare Group, Atasehir Hospital, Istanbul, Turkey; 5 Department of Pediatric Nephrology, Faculty of Medicine, Çukurova University, Adana, Turkey; 6 Néphrologie Pédiatrique, Institut Imagine, Hôpital Universitaire Necker-Enfants Malades, Assistance Publique – Hôpitaux de, INSERM U1163, Université Paris Cité , Paris, France; 7 Department of Pediatric Nephrology and Hypertension, Chair of Pediatrics, Institute of Pediatrics, Jagiellonian University Medical College , Wielicka 265 St, Cracow, 30-663 Poland; 8 Department of Paediatric Nephrology, Great Ormond Street Hospitaland , UCL Institute of Child Health, London, UK; 9 Pediatric Nephrology, Nottingham Children’s Hospital, Nottingham, UK; 10 Pediatric Nephrology, Rheumatology and Dermatology Unit, Est Medical School, University, Lyon Hospices Civils de Lyon, Lyon 1Lyon, France; 11 Pediatric Nephrology Unit, Bordeaux Children’s University Hospital, University of Bordeaux, Bordeaux, France; 12 Pediatric Nephrology Unit, 1, Pediatric Department, Aristotle University, Hippokration General Hospital, Thessaloniki, Greece; 13 Department of Renal Medicine, University College London Centre for Kidney and Bladder Health, London, UK; 14 Department of Women’s and Children’s Health, University of Liverpool, Liverpool, UK; 15 Schneider Children’s Medical Center Israel, Gray Faculty of Health Sciences, Tel Aviv University, Tel Aviv, Israel; 16 Pediatric Nephrology, Bristol Children’s Hospital, Bristol, UK; 17 Department for Pediatric Kidney, Metabolic and Neurological Diseases, Hannover Medical School, LiverHannover, Germany; 18 Division of Pediatric Nephrology, University Medical Center Hamburg/Eppendorf, Hamburg, Germany; 19 Division of Pediatric Nephrology, Emma Children’s Hospital, Amsterdam University Medical Center, Amsterdam, The Netherlands

**Keywords:** Pediatric Nephrology, Education, Training, Board Examination, Europe

## Abstract

**Background:**

Subspecialty recognition and certification in Pediatric Nephrology remain heterogeneous across Europe and globally, resulting in variations in training standards and professional mobility. To address this gap, the European Society for Paediatric Nephrology (ESPN) launched the European Board Certification in 2020 as a pan-European and curriculum-based assessment of knowledge and clinical reasoning in Pediatric Nephrology.

**Methods:**

We performed a descriptive and analytical evaluation of candidates who applied for the ESPN Board Certification in Pediatric Nephrology between 2020 and 2025. Candidate characteristics, examination performance, and factors associated with success were analyzed. The exam consisted of 100 case-based multiple-choice questions developed according to a predefined blueprint. Multivariable logistic regression was used to identify factors independently associated with passing. Additionally, a cross-sectional survey assessed candidates’ perceptions of the examination and its professional impact.

**Results:**

A total of 349 pediatric nephrologists from 52 countries and four continents completed the examination. Median age was 38.2 years (IQR 34.7–43.5), and 59.9% were female. The overall pass rate was 54.7%, with significant variation by continent (*p* = 0.014). Attendance at the International Pediatric Nephrology Association (IPNA)-ESPN Junior Master Class was associated with higher odds of success (OR 3.76, 95% CI 1.65–5.87, *p* < 0.001), whereas increasing age was associated with lower odds of success (OR 0.66 per additional year, 95% CI 0.55–0.78, *p* = 0.002). Among 74 survey respondents, perceptions regarding the examination were highly positive: 93.2% considered the content clinically relevant, 89.2% reported increased self-confidence, and 75.6% perceived a positive professional impact.

**Conclusions:**

Since its launch, the ESPN Board Examination has emerged as an internationally acknowledged benchmark of key knowledge and clinical reasoning in Pediatric Nephrology. Structured preparatory education enhances examination success, and certification is widely perceived as professionally meaningful. The examination plays a vital role in standardizing subspecialty criteria and reinforcing the professional identity within Pediatric Nephrology.

**Graphical abstract:**

**Figure:**
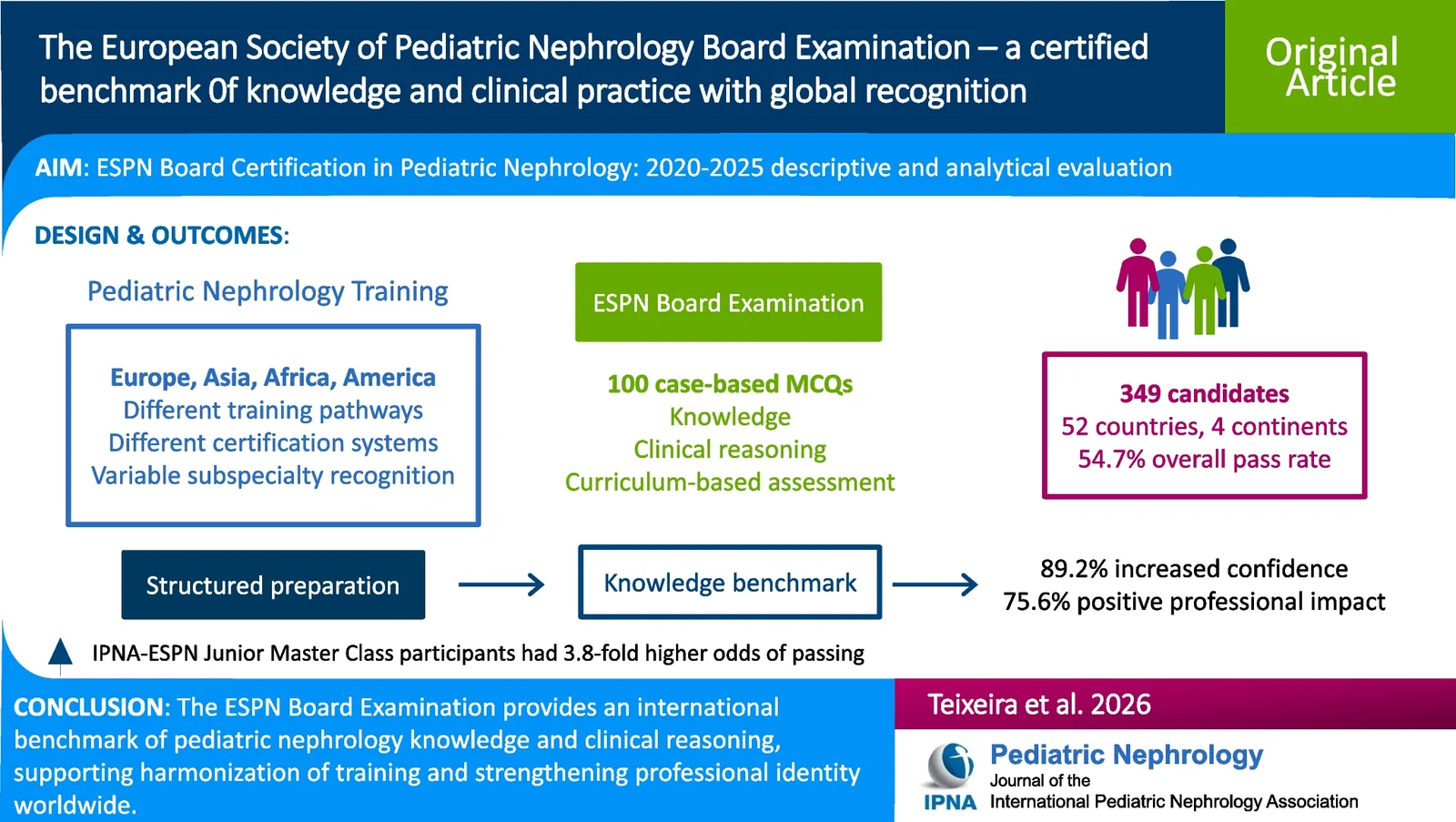
A higher resolution version of the Graphical abstract is available as Supplementary information

**Supplementary Information:**

The online version contains supplementary material available at https://doi.org/10.1007/s00467-026-07436-x.

## Introduction

Pediatric Nephrology is a highly specialized discipline that encompasses complex diagnostic reasoning, advanced therapeutics, and long-term multidisciplinary care. Despite its clinical and academic maturity, the formal recognition of Pediatric Nephrology as a subspecialty remains heterogeneous across Europe and worldwide. In several countries, structured training pathways exist; however, they do not always have international recognizable standards, which creates disparities in certification, professional mobility, and workforce development [1–4].

The absence of uniform subspecialty recognition has practical consequences. Trainees may complete extensive additional training without formal certification, while health systems lack standardized benchmarks for competence. This variability also complicates cross-border professional mobility and undermines efforts to harmonize pediatric kidney care across regions [5, 6].

Within this context, building on the success of the IPNA-ESPN Junior Master Class (JMC), the ESPN Board Examination was established as an independent, knowledge-based assessment aligned with the ESPN training curriculum [7]. Introduced in 2020, the examination was conceived not as a replacement for national accreditation systems, but as a complementary, supranational benchmark aiming to standardize the evaluation of physicians who have completed training in Pediatric Nephrology, and to serve as a quality label of key knowledge and clinical reasoning expected of a trained pediatric nephrologist.

From an organizational perspective, one of the central challenges has been ensuring that the examination remains inclusive of diverse training environments whilst maintaining a consistent standard. This required careful blueprinting, regular question review, expert external support, and ongoing alignment with evolving clinical practice [8], while also necessitating an approach that is standardized yet sensitive to international differences in clinical practice, healthcare systems, and available resources.

## Methods

### Status of the ESPN Board Examination

The exam consists of 100 case-based multiple-choice questions covering all aspects of Pediatric Nephrology, namely congenital anomalies of the kidney and urinary tract (CAKUT), bladder dysfunction, glomerular disorders, tubulopathies and inherited diseases, fluid and electrolyte balance, stone disease, acute kidney injury and chronic kidney disease, dialysis and transplantation, psychosocial issues, transition and statistics, according to a pre-defined blueprint developed by the ESPN Education Committee [8], proportionally reflecting the relative clinical relevance and training emphasis of major curriculum domains. The examination contains representation from all core syllabus areas and multiple competency domains including health management and prevention, disease mechanism, diagnosis, and disease management, as shown in Table 1. This same curriculum is fully covered by the IPNA-ESPN JMC, an educational activity especially designed for pediatricians with special interest in Pediatric Nephrology and early career pediatric nephrologists, a joint organization between ESPN and IPNA ongoing since 2014 [9]. All questions are peer reviewed and contain a validation comment justifying the correct answer and references to topical literature, specifically ESPN and IPNA current guidelines [10, 11], Pediatric Nephrology Journal educational reviews [12] and Pediatric Nephrology textbook [13].

**Table 1:** ESPN Board examination blueprint of multiple-choice questions

Topic	Health maintenance (prevention)	Mechanism (pathophysiology)	Diagnosis	Management
CAKUT/bladder dysfunction (including UTI, enuresis, spinal problems; embryology, antenatal diagnosis and radiology)	1	2	9	9
Glomerular diseases (including SSNS and SRNS, CNS, vasculitis/lupus/HUS/C3 GN; histopathology)		2	8	9
Inherited disorders (including tubular diseases, cystic kidney disease, nephronophthisis, genetic and antenatal diagnosis)		2	8	5
Fluid and electrolyte balance, AKI		1	2	3
Stones		1	1	1
Chronic kidney disease (including measuring (e)GFR, nephroprotection, nutrition, anemia, growth, hypertension)	1	2	3	9
Dialysis (including acute dialysis)	1	1	3	7
Transplantation		1	2	2
Psychological issues and transition	1			1
Biostatistics				2
Total	4	12	36	48

*CAKUT* congenital anomalies of the kidney and urinary tract, *UTI* urinary tract infection, *SSNS* steroid sensitive nephrotic syndrome, *SRNS* steroid resistant nephrotic syndrome, *CNS* congenital nephrotic syndrome, *HUS* hemolytic uremic syndrome, *GN* glomerulopathy, *AKI* acute kidney injury, *GFR* glomerular filtration rate

The evaluation of adequate skills and attitudes of the applicants is performed before the examination, based on the duration of training in a tertiary Pediatric Nephrology center and a letter of its training director certifying the eligibility of the candidate.

The candidates’ application requires a registration fee to support the technical and organizational aspects of the evaluation and certification process.

### ESPN education committee

The ESPN Board Examination preparation, planning, implementation, and evaluation is the responsibility of the ESPN Education Committee, composed of elected representatives from different European regions, appointed by the ESPN council [8]. All ESPN Education Committee members are specifically trained on the methodology of professional postgraduate assessment [14], organized, and monitored by the ESPN council. Committee participation is voluntary, and membership duration follows the governance regulations established by ESPN.

### Candidates’ eligibility

All candidates are required to submit their medical and pediatrics or nephrology specialty certificate and confirm their adequate training in Pediatric Nephrology (a minimal duration of clinical experience in the field of at least 24 months). A letter from the Pediatric Nephrology training director confirming start and end dates of training, adequate clinical skills at the tertiary level, and a good quality professional attitude towards patients, families, and colleagues is also mandatory.

### Assessment method

The ESPN Board Examination is conducted in English and performed online via the remotely proctored professional *Examfolio* platform (Ektimo ApS Gamm eltoftsgade 12B, kld. 1353 Copenhagen K, DE) [15]. The exam has a maximum duration of 150 min, and all candidates are individually monitored in real time by members of the ESPN Education Committee. Use of external resources, including artificial intelligence tools, online resources, textbooks, or communication with third parties, is strictly prohibited during the examination. All candidates complete the same 100-question examination in a given year, based on the predefined curriculum-aligned blueprint.

*Examfolio* platform allows an electronic score calculation based on the pre-defined correct answer to each question. The final score and pass-fail criteria are defined by the ESPN Education Committee, based on the *Angoff* method [16] and the evaluation of all questions’ performance is assessed after the exam, concerning their level of difficulty, the degree of discriminative power and the general reliability (internal consistency) of the assessment. ESPN Board Examination metrics are shown in Table 2. KR20 coefficient indicates whether the exam discriminates among students who master the subject matter and those who do not. KR20 coefficients close to 1 show that the exam discriminates well between high and low-performing candidates, whereas KR20 close to 0 shows that it does not. KR20 > 0.7 is usually accepted as an exam of sufficient quality. The exam results (both numerical score and pass/fail information) are individually communicated to each candidate, providing an opportunity to discuss the outcome with an education committee member who can provide career advice as required.

**Table 2:** ESPN Board Certification Exam’s metrics (2020–2025)

Year	Number of participants	Lowest score	Highest score	Mean score	KR 20 coefficient	Pass mark	Number (percentage) of participants who passed
2020	96	45	89	72	0.74	68	64 (67)
2021	74	42	82	60	0.73	63	31 (42)
2022	37	25	78	57	0.83	58	20 (54)
2023	47	40	80	59	0.72	60	22 (47)
2024	45	21	83	59	0.85	60	23 (51)
2025	50	23	85	62	0.88	60	31 (62)
2020–2025 average	58	33	83	61	0.79	61	32 (54)

The candidates that successfully pass the exam receive the ESPN Board Certification in Pediatric Nephrology diploma.

### Statistical analysis

This study statistical analysis was performed using SPSS Statistics 30.0 (IBM Corp., Armonk, NY, USA). Statistical significance was defined as *p* < 0.05.

Descriptive statistics were used to summarize candidate characteristics. Continuous variables are presented as median and interquartile range (IQR), and categorical variables as counts and percentages.

Comparisons of examination outcomes across continents were performed using the chi-square (*χ*^2^) test to assess associations between categorical variables.

To identify independent factors associated with examination approval, multivariable logistic regression analysis was conducted. Variables entered into the model included sex, age, year of examination, continent of origin, and JMC attendance. Results are reported as odds ratios (ORs) with 95% confidence intervals (CIs).

## Results

### Participants and outcomes

Since 2020, a total of 349 fully trained pediatric nephrologists applied for the ESPN Board Certification in Pediatric Nephrology. The median age of candidates was 38.2 years (IQR 34.7–43.5), and 209 were female (59.9%). The highest number of participants was recorded in 2020 (n = 96).

**Fig. 1:**
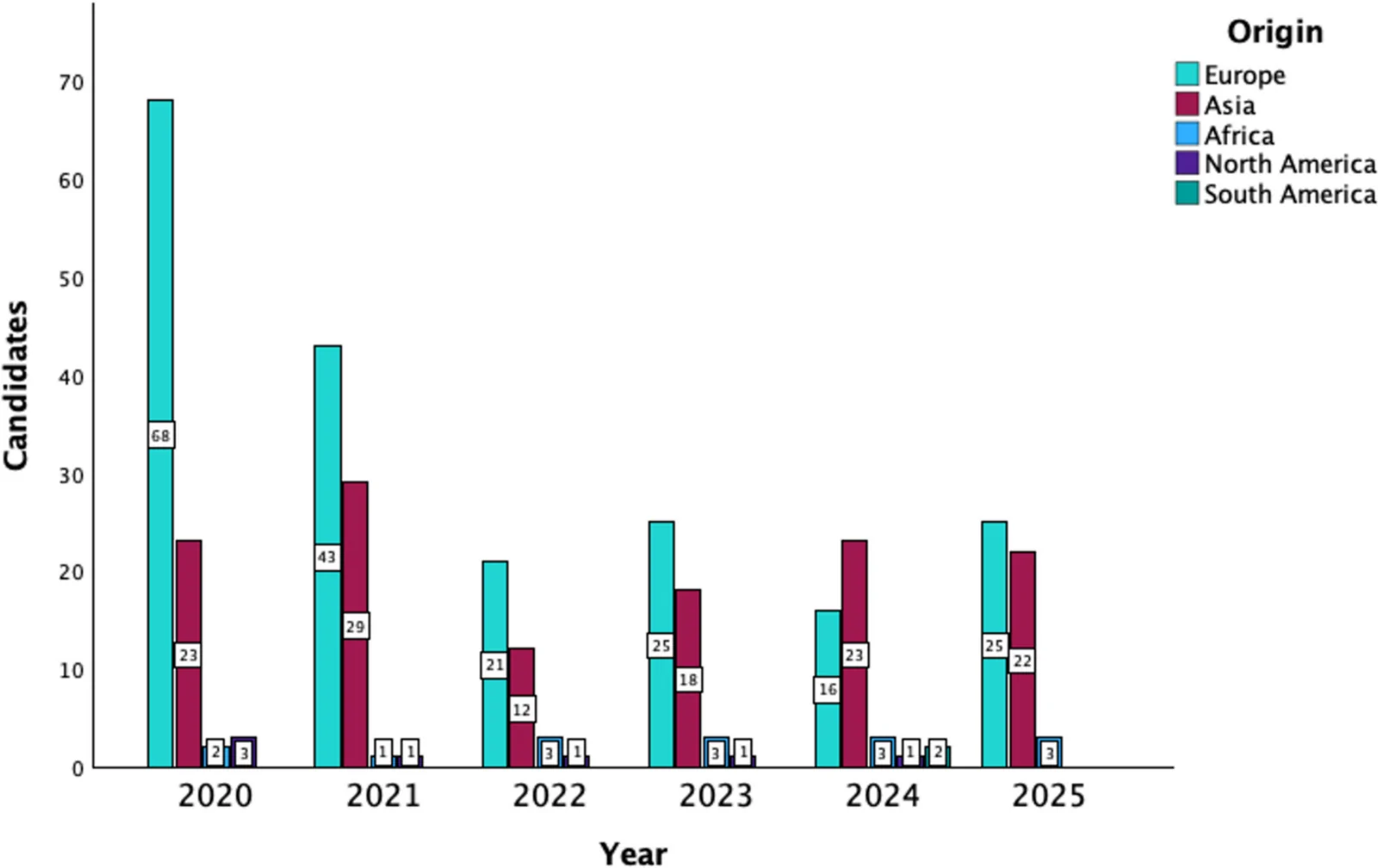
ESPN Board Exam candidates’ distribution by year and continent

Participants came from 52 countries, as shown in Table 3. Regarding continent of origin, most candidates were from Europe (56.7%), followed by Asia (36.4%), Africa (4.3%), and America (2.6%; 2.0% North America and 0.6% South America). In 2024, Asian candidates represented the majority (51.1%), and, for the first time, candidates from South America participated. The distribution of candidates by year and continent is shown in Fig. 1.

**Table 3:** ESPN Board Exam Candidates’ country of origin

Belgium, Bulgaria, Canada, Cote d'Ivoire, Croatia, Czech Republic, Denmark, Egypt, Finland, France, Georgia, Germany, Greece, Guyana, Hong Kong, India, Indonesia, Iraq, Italy, Japan, Jordan, Kenya, Kuwait, Lebanon, Malaysia, Mexico, Montenegro, Morocco, Netherlands, Oman, Pakistan, Palestine occupied territory, Poland, Portugal, Qatar, Romania, Saudi Arabia, Serbia, Singapore, South Africa, Spain, Sri Lanka, Sudan, Sweden, Switzerland, Thailand, Tunisia, Turkey, United Arab Emirates, UK, USA, Yemen

Candidates applied for the examination a median of 2.0 years (IQR 0.6–4.3) after becoming eligible (defined as completion of at least 24 months of Pediatric Nephrology practice in their home country, certified by a training director from a tertiary center). Overall, 210 candidates (60.2%) were former IPNA–ESPN JMC participants.

**Fig. 2:**
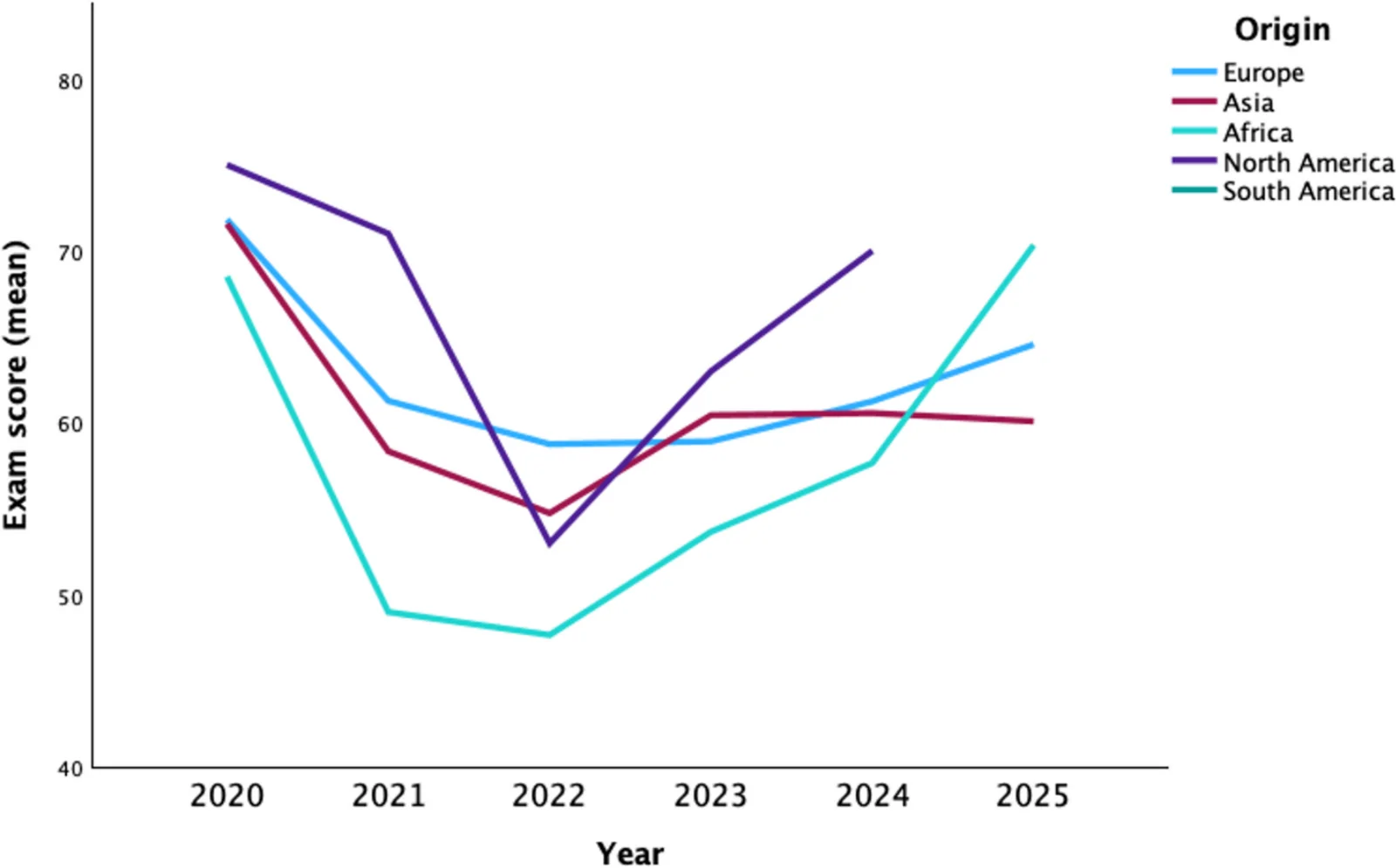
ESPN Board Exam score (mean) by year and continent

Across the study period, the median examination score was 64/100 (IQR 56–71), and the overall pass rate was 54.7%. Pass rates differed by continent: 54.3% in Asia (69/127), 53.3% in Africa (8/15), and 39.4% in Europe (78/198). The association between continent and examination outcome was statistically significant (*χ*^2^ test, *p* = 0.014). Mean examination scores by continent over time are presented in Fig. 2.

**Fig. 3:**
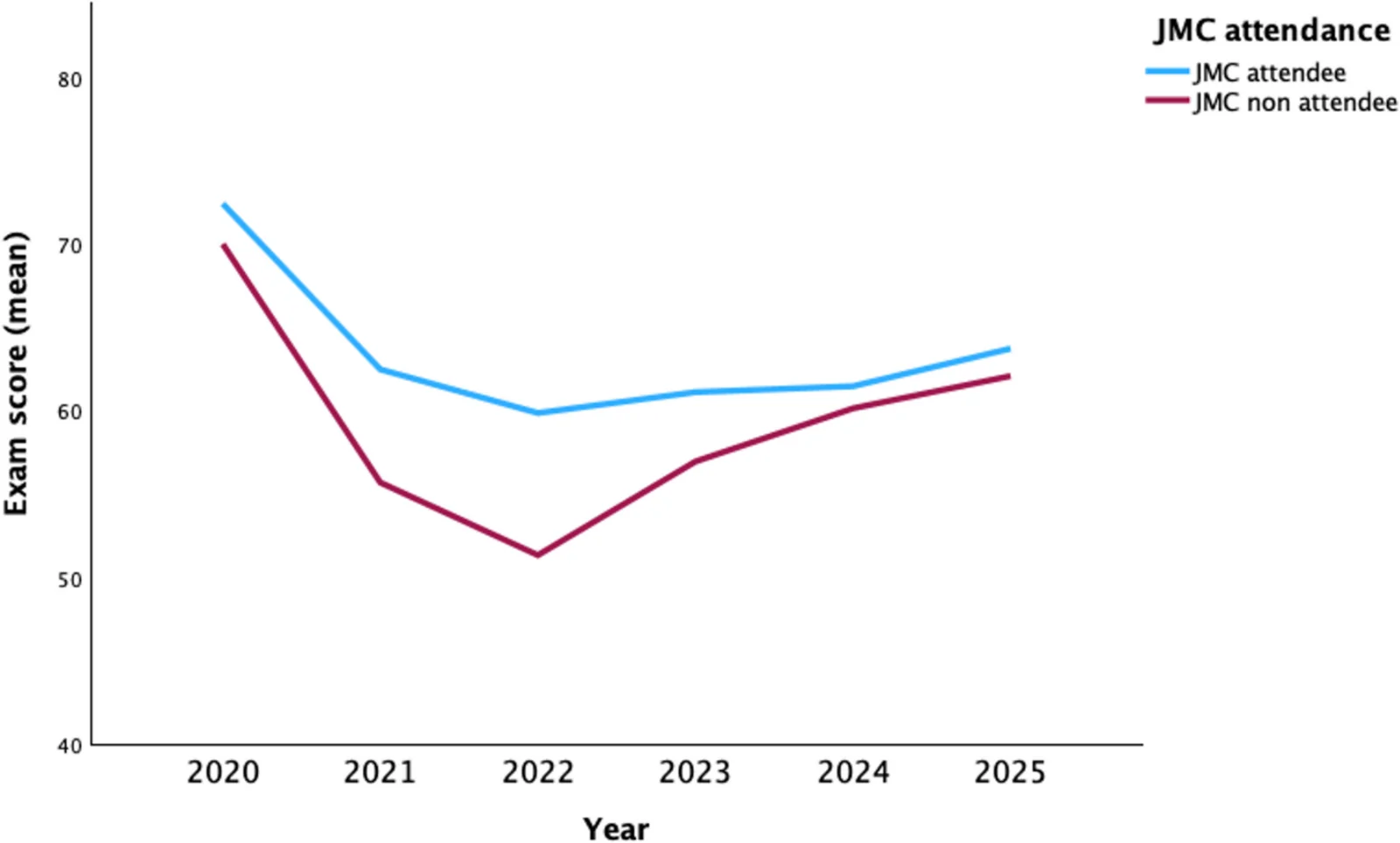
ESPN Board Exam score (mean) and JMC attendance

On multivariable logistic regression analysis, JMC attendance was independently associated with higher odds of passing the examination (OR = 3.76, 95% CI 1.65–5.87, *p* < 0.001). Increasing age was independently associated with lower odds of passing, corresponding to a 34% reduction in odds per additional year of age (OR = 0.66, 95% CI 0.55–0.78, *p* = 0.002). Mean examination scores according to JMC attendance over time are shown in Fig. 3.

### Participants’ feedback evaluation

With the growing recognition of the ESPN Board Certification, we hypothesized that examination success and certification would translate into measurable career advancement and increased professional confidence. Despite that, data on candidates’ perceptions, preparation strategies, and professional impact were limited.

To address these questions, we conducted a cross-sectional survey among our candidates. The survey was designed to explore candidates’ experiences with the examination, including perceptions of its organization, relevance to clinical practice, and level of difficulty. Furthermore, we aimed to assess the perceived professional impact of certification, factors associated with examination success and career-related outcomes.

A total of 74 candidates from 37 countries and four continents responded to the survey (21%). The median age of participants was 41.5 years (IQR 38–47), and 71.6% were female. National certification in Pediatric Nephrology was available in 16 countries (43.2%), corresponding to 64.8% (n = 48) of respondents.

More than half of the participants (55.6%) first undertook the examination in 2020 or 2021, and 14.9% reported repeating the exam after an initial unsuccessful attempt.

Perceptions of the examination were very positive. All participants rated the organization of the exam above 7/10. The majority considered the content highly relevant to their clinical practice (93.2%), and 89.1% reported that the level of difficulty met their expectations.

Regarding preparation, 85.1% considered the recommended and available resources adequate, and 68.3% reported a preparation period of approximately 3 months.

In terms of professional impact, 59.5% reported that the ESPN Board Certification in Pediatric Nephrology had a meaningful impact on their career, and 50% felt it contributed to their professional growth. A minority reported objective career advancement, with 8% receiving a promotion and 6.8% obtaining a salary increase following certification. Self-confidence improved in 89.2% of respondents, and 75.6% perceived an improvement in their clinical performance. Overall, 75.6% reported a positive impact of the certification on their professional life.

Survey respondents were broadly comparable to the overall examination cohort regarding sex distribution and JMC attendance; however, respondents were older and more frequently successful at the examination, suggesting potential response bias toward candidates with more favorable perceptions of the certification process.

## Discussion

This report provides a comprehensive overview of the implementation and performance of the ESPN Board Examination in Pediatric Nephrology since its launch in 2020, as well as candidates’ perceptions of its educational and professional impact. The findings demonstrate growing international engagement, moderate overall pass rates, and significant geographic variation. Participation in the IPNA-ESPN JMC was independently associated with examination success, while certification was widely perceived as professionally meaningful, particularly in terms of increased confidence and clinical performance.

Subspecialty recognition in Pediatric Nephrology remains heterogeneous across Europe and globally. In this context, the ESPN Board Examination represents an international benchmark aligned with a standardized curriculum. The steady increase in applications, including participation from four continents and the growing representation of Asian candidates in recent years, suggests that the certification is gaining international credibility. Beyond serving as a knowledge-based assessment, the examination may contribute to harmonizing training expectations across diverse educational systems and support professional mobility in regions where national certification pathways are variable or absent.

The association between JMC attendance and higher odds of passing the examination represents one of the most relevant findings. The JMC is a structured educational initiative focused on case-based discussion and guideline-oriented management. Its independent predictive value supports the importance of structured, curriculum-aligned preparation in subspecialty education. These findings suggest that harmonized educational programs may contribute to reducing variability in knowledge acquisition across training environments. In addition, this structured educational activity may serve as a model for other subspecialties seeking to harmonize training standards across regions.

Increasing age was associated with lower examination success. This finding should be interpreted cautiously. The association likely reflects factors such as greater temporal distance from structured training, increased clinical and administrative responsibilities, or even reduced familiarity with computer-based multiple-choice testing formats. Importantly, examination performance represents a measure of knowledge and clinical reasoning aligned with a defined curriculum and does not directly equate to overall professional capability.

Survey responses indicated that the examination was perceived as well organized, clinically relevant, and appropriately challenging. Most respondents considered the recommended preparation resources adequate. While objective career advancement following certification was limited, a substantial proportion reported increased self-confidence and perceived improvement in clinical performance. These findings suggest that the examination may contribute not only to external validation of knowledge but also to professional development and reinforcement of subspecialty identity. In regions where Pediatric Nephrology lacks formal regulatory recognition, such certification may provide important symbolic and practical reinforcement of subspecialty expertise. However, these findings should be interpreted with caution, given the possibility of response bias toward candidates with more favorable examination outcomes.

From an organizational perspective, the ESPN Board Examination is grounded in a predefined blueprint aligned with the official curriculum, employs peer-reviewed case-based questions, and applies the *Angoff* method for standard setting. The overall pass rate should be interpreted within the context of an international examination involving candidates from heterogeneous training systems with varying levels of structured subspecialty preparation. The examination was intentionally designed as a high-standard assessment emphasizing applied clinical reasoning rather than factual recall alone. Post-examination psychometric evaluation, including assessment of question difficulty, discrimination indices, and internal consistency, further supports the methodological robustness of the assessment process. The stability of KR20 coefficients over time, with an average of 0.79, supports the reliability and appropriate discriminatory capacity of the examination, while ongoing annual psychometric evaluation contributes to continuous quality assurance. These elements are essential to maintaining credibility, fairness, and transparency in high-stakes certification examinations.

Beyond its role as an assessment tool, the ESPN Board Examination may also influence the educational landscape of Pediatric Nephrology training. Curriculum-based certification assessments often shape learning priorities, encourage structured and guideline-oriented preparation, and promote harmonization of training expectations across institutions and countries. In this context, the examination may contribute not only to benchmarking specialist knowledge and clinical reasoning, but also to reinforcing competency-based educational frameworks and reducing variability in subspecialty training environments.

While several pediatric subspecialties have developed European or multinational examinations aimed at standardizing knowledge assessment and harmonizing training, formal international board certification processes remain relatively uncommon. Most existing initiatives focus on establishing shared curricula or structured examinations intended for benchmarking rather than conferring a globally recognized certification. Notable examples include the European Board of Paediatric Surgery (EBPS), which administers a two-part examination (written and oral), successful completion of which confers the title of Fellow of the European Board of Paediatric Surgery (FEBPS) [17]. Similarly, the International Gynecologic Cancer Society Global Fellowship program incorporates structured educational and assessment components, although it does not constitute a fully standardized international board certification process [18]. Within this context, pediatric nephrology appears to be at the forefront of international collaboration in developing a structured and standardized certification process with formalized assessment and oversight mechanisms.

## Conclusions and future directions

The ESPN Board Examination provides a structured, curriculum-based assessment aligned with contemporary standards in Pediatric Nephrology. Since its introduction in 2020, it has attracted increasing international participation and is progressively recognized as a benchmark of key knowledge and clinical reasoning within the subspecialty. Participation in structured preparatory initiatives appears to enhance examination success, and certification is widely perceived as professionally meaningful.

Ensuring inclusivity while maintaining consistent standards remains an ongoing challenge. Language barriers and differing familiarity with multiple-choice testing formats may influence performance. Future considerations could include expanded preparatory resources or exploration of multilingual support, while carefully preserving the integrity and comparability of the assessment.

In regions where Pediatric Nephrology lacks formal regulatory recognition, the ESPN Board Examination may serve an additional role while providing external validation of subspecialty-level competence. While examinations alone cannot substitute for legislative or regulatory reform, they may contribute to professional identity formation and support advocacy efforts for formal recognition.

Future studies assessing professional mobility, academic progression, leadership opportunities, and institutional recognition may further clarify the broader impact of international subspecialty certification in Pediatric Nephrology.

Continued evaluation of its educational impact and long-term outcomes, collaboration between professional societies, training centers and regulatory bodies will be essential to further strengthen harmonization of Pediatric Nephrology training and ultimately ensure high standards of care for children with kidney disease.

## Supplementary Information

Below is the link to the electronic supplementary material.

Graphical abstract (PPTX 76.8 KB)

## Data Availability

The datasets generated and analysed during the current study are not publicly available due to examination security and confidentiality requirements.

## Declarations

### The ESPN education committee

Chair: Elena Levtchenko (The Netherlands).

Members: Dieter Haffner and Jun Oh (Germany), Rezan Topaloglu and Aysun Karabay Bayazit (Turkey), Ana Teixeira (Portugal), Olivia Boyer, Justine Bacchetta and Jerome Harambat (France), Stella Stabouli and Nikoleta Printza (Greece), Dorota Drozdz (Poland), Zainab Arslan, Louise Oni, Caroline Platt and Rukshana Shroff (UK) and Daniella Levy Erez (Israel).
